# Results of treatment with alemtuzumab in a Spanish cohort of patients with multiple sclerosis in the real world: The RealMS study

**DOI:** 10.3389/fneur.2023.1112193

**Published:** 2023-03-14

**Authors:** Sara Eichau, Rocío López Ruiz, María Ruíz de Arcos, Juan Luis Ruiz-Peña, Guillermo Navarro, Miguel Ángel Calleja, José Luis Moreno-Amador, Julio Dotor García-Soto

**Affiliations:** ^1^Multiple Sclerosis Unit, Neurology Service, Hospital Universitario Virgen Macarena, Seville, Spain; ^2^Department of Pharmacy, Hospital Universitario Virgen Macarena, Seville, Spain; ^3^Multiple Sclerosis Unit, Sanofi, Barcelona, Spain

**Keywords:** real-world data, multiple sclerosis, alemtuzumab, disease-modifying therapy, effectiveness, safety

## Abstract

**Background:**

Alemtuzumab (ALZ) is a humanized monoclonal antibody approved for the treatment of patients with highly active relapsing-remitting multiple sclerosis (RRMS) administered in two annual courses. The objective of this study was to describe the effectiveness and safety data of ALZ and to report the health resource utilization in patients receiving this treatment.

**Methods:**

In this retrospective, non-interventional study, information was retrieved from patients' medical charts at one center in Spain. Included patients were ≥18 years old, and ALZ treatment was initiated between 1 March 2015 and 31 March 2019, according to routine clinical practice and local labeling.

**Results:**

Of 123 patients, 78% were women. The mean (standard deviation, SD) age of patients at diagnosis was 40.3 (9.1) years, and the mean time since diagnosis was 13.8 (7.3) years. Patients were previously treated with a median (interquartile range; IQR) number of two (2.0–3.0) disease-modifying treatments (DMTs). Patients were treated with ALZ for a mean (SD) of 29.7 (13.8) months. ALZ reduced the annualized relapse rate (ARR) (1.5 before vs. 0.05 after; *p* < 0.001) and improved the median EDSS (4.63 before vs. 4.00 after; *p* < 0.001). Most (90.2%) patients were relapse-free while receiving ALZ. The mean number of gadolinium-enhancing [Gd+] T1 lesions was reduced (1.7 before vs. 0.1 after; *p* < 0.001), and the mean number of T2 hyperintense lesions was maintained (35.7 before vs. 35.4 after; *p* = 0.392). A total of 27 (21.9%) patients reported 29 autoimmune diseases: hyperthyroidism (12), hypothyroidism (11), idiopathic thrombocytopenic purpura (ITP) (3), alopecia areata (1), chronic urticaria (1), and vitiligo (1). The mean number of health resources (outpatient visits, emergency room visits, hospital admissions, and tests performed in the hospital) used while patients were treated with ALZ progressively decreased from year 1 to year 4, except for a slight increase at year 2 of outpatient visits.

**Conclusion:**

The ReaLMS study provides real-world evidence that ALZ can promote clinical and magnetic resonance imaging disease remission, as well as disability improvement in patients with MS, despite several prior DMT failures. The ALZ safety profile was consistent with data available from clinical trials and other real-world studies. Healthcare resource use was reduced throughout the treatment period.

## Introduction

Multiple sclerosis (MS) is a chronic, inflammatory, demyelinating, and neurodegenerative disease that affects at least 2.8 million people worldwide. It is the leading non-traumatic cause of neurological disability in young adults (1). MS has a negative impact on the health-related quality of life (HRQoL) among all patients (2) and entails considerable losses of productivity and unemployment among patients of working age (3). Work productivity losses together with healthcare resource utilization impose an important economic burden not only on patients themselves but also on society (4).

The advent of newer and more efficacious disease-modifying treatments (DMTs) has increased the therapeutic arsenal for MS, bringing new opportunities for individualized treatment. Alemtuzumab (ALZ) (Lemtrada^®^) is a humanized monoclonal antibody approved for the treatment of patients with highly active relapsing-remitting MS that is given as a pulsed immune reconstitution therapy usually in two treatment cycles. The phase III clinical trials, CARE-MS I (5) and CARE-MS II (6), showed that patients treated with ALZ had greater improvements in clinical and radiological outcomes compared to patients treated with interferon beta-1a (SC IFNB-1a; Rebif^®^). Improvements in clinical outcomes were observed in both treatment-naive patients (5) and non-responders to previous treatment (6) and included a significant reduction in relapse and confirmed disability progression. The results of ALZ efficacy persisted over time and were maintained over 12 years, with a safety profile consistent with prior studies (7, 8). Extension studies also observed reductions in the rate of brain volume loss, with a high proportion of patients achieving no evidence of disease activity (NEDA) over time (7–9) and improvements in HRQoL (10). Due to reports of rare, but serious, adverse events after ALZ authorization, the indication was amended to restrict ALZ use to patients with highly active RRMS after at least one previous DMT or with rapidly evolving severe RRMS (11).

Patients with MS treated with ALZ in the clinical setting are usually older, have longer average disease duration, and have a higher disability than patients from clinical trials (12, 13). The differences in demographic and clinical characteristics among patients in clinical trials and real-world settings highlight the need to evaluate outcomes with ALZ in a broader and more heterogeneous population. To date, data on the effectiveness and safety profile of ALZ from observational studies are limited (14–21), and studies that provide information on ALZ effectiveness and safety together with the use of healthcare resource associated with these patients are lacking. Given that real-world data are essential for gathering information on a wider spectrum of patients and, therefore, improving individual patient management, the purpose of this study is to provide data on the effectiveness and safety of ALZ and the healthcare resource use of patients with MS in the clinical practice setting.

## Materials and methods

### Study design and patients

ReaLMS was a retrospective, single-center, non-interventional study conducted at the Multiple Sclerosis Unit of the Neurology Department of the Virgen Macarena University Hospital in Seville (Spain). Patient information was retrospectively retrieved from their medical charts, when available.

Eligible patients were aged ≥18, had MS meeting McDonald criteria (22, 23), and initiated ALZ treatment between 1 March 2015 and 31 March 2019, according to routine clinical practice and local labeling. Patients were excluded if they had received ALZ as part of a clinical trial. Patient data were registered (database cutoff) until 5 March 2020.

### Study objectives and assessments

Effectiveness was assessed after at least 1 year of ALZ treatment by the following clinical outcomes: annualized relapse rate (ARR) before and after ALZ treatment, time until first relapse, percentage of relapse-free patients, Expanded Disability Status Scale (EDSS) score before and after ALZ treatment (last score available), and the percentage of patients with cumulative 6-month confirmed disability worsening (CDW) and cumulative 6-month confirmed disability improvement (CDI). A relapse was defined as new or worsening neurological symptoms attributable to MS, lasting for at least 48 h, without pyrexia, after at least 30 days of clinical stability, with an objective change on neurological examination. The ARR was calculated as the number of relapses per patient year. CDW was defined as a ≥1-point EDSS score increase (≥1.5 if baseline EDSS = 0) and CDI as a ≥1.0-point decrease from the core study baseline EDSS score, assessed in patients with baseline EDSS scores ≥2.0). The EDSS was assessed after at least 30 days from the onset of the last relapse.

Secondary assessments included demographics (sex and age) and clinical characteristics (MS type, disease duration, time since ALZ initiation, and prior MS treatments), MRI disease activity (gadolinium-enhancing [Gd^+^] lesions and new T2-hyperintense lesions), resources use (MS-related symptomatic treatments administered after ALZ, outpatient visits, admissions to emergency rooms, hospital admissions, single day admissions, additional tests performed, the need of additional courses of ALZ, and days of incapacity for work), and the frequency and characterization of main adverse events (AEs) and infusion-associated reactions (IARs).

### Standard protocol approvals, registrations, and patient consent

Written informed consent regarding the use of patients' medical data for research purposes was obtained from all patients. The study was approved by the ethics committee of the Hospital Universitario Virgen Macarena. The study was performed in accordance with the International Conference on Harmonization Guidelines for Good Clinical Practice, the Declaration of Helsinki, and the Spanish legislation for post-authorization studies.

### Statistical analysis

The description of quantitative variables was performed by using the measures of central tendency and dispersion (mean [standard deviation, SD]) or median [interquartile range, IQR] values). For the description of qualitative variables, absolute and relative frequencies were used. For relative frequencies, two percentages were calculated: the total percentage, which was the percentage of the sum of valid responses and missing values, and the valid percentage, which was the percentage of the total valid responses. Changes in the ARR, EDSS, and MRI were calculated by using the non-parametric Wilcoxon test. The Kaplan–Meier method (95% confidence interval [CI]) was used to calculate the time-to-first relapse after ALZ initiation. The *p*-value of < 0.05 was considered statistically significant. No imputations for missing data were performed. All statistical analyses were performed by using Statistical Package for Social Sciences (SPSS) version 22.

Power and sample size calculations were undertaken. A population sample of 121 subjects was estimated to be required to detect clinical outcomes that were present in at least 10% of the study population, accepting an alpha risk of 0.05 for a precision of ±5.5 percentage units in a two-sided contrast for an estimated proportion of 10%. A replacement rate of 5% was considered.

## Results

### Demographic and clinical characteristics

A total of 123 patients met the selection criteria and were included in the study. The baseline characteristics of the patients are summarized in Table 1. Most patients were women (78%) and had a mean (SD) age of 40.3 (9.1) years (ranging from 20 to 64 years) during diagnosis. Before ALZ initiation, patients had a median (IQR) EDSS score of 4.6 (3.5–6.0) and an ARR of 1.5 in the previous year. All patients had active disease with or without underlying progression: 104 (85.4%) patients had RRMS, 17 (13.8%) had secondary progressive MS (SPMS), and one (0.8%) had primary progressive (PPMS). The median number of previous DMTs was 2 (2.0–3.0): 11 (8.9%) patients were treatment-naive, 97 (78.8%) received ≥2 previous DMTs, and 53 (43%) received ≥3 previous DMTs. The DMTs that most frequently received prior ALZ were fingolimod (50.9%), dimethyl fumarate (12.5%), natalizumab (11.6%), and glatiramer acetate (4.5%). Reasons for switching from the previous DMT to ALZ were the lack of effectiveness (83%), safety reasons including the seroconversion of JC virus and treatment intolerance (9%), and being the first DMT due to aggressive MS (8%). At data collection, 23 patients (18.7%) had received one ALZ course, 92 patients (74.8%) had received two courses, seven patients (5.7%) had received three courses, and one patient (0.8%) had received four courses. Those patients who had received only one course in data collection, received the second course later, according to clinical practice.

**Table 1:** Baseline characteristics.

**Variable**	**Value (*N* = 123)**
Age at MS diagnosis (years), mean (SD)	40.3 (9.1)
Gender (female), n (%)	96 (78)
Time from diagnosis (years), mean (SD)	13.8 (7.3)
Number of relapses in the previous year, mean (SD)	1.5 (0.8)
EDSS score, median (IQR)	4.5 (3.5–6.0)
Number of T1 Gd + lesions^a^, mean (SD)	1.7 (3.3)
Number of T2-lesions^a^, mean (SD)	35.5 (23.6)^b^
Number of previous DMT, *n* (%)	
0	11 (8.9)
1	15 (12.2)
2	44 (35.8)
3	40 (32.5)
4	8 (6.5)
5	3 (2.4)
6	2 (1.6)

a In the MRI scan performed before alemtuzumab initiation;

b N = 122.

### Effectiveness

**Figure 1:**
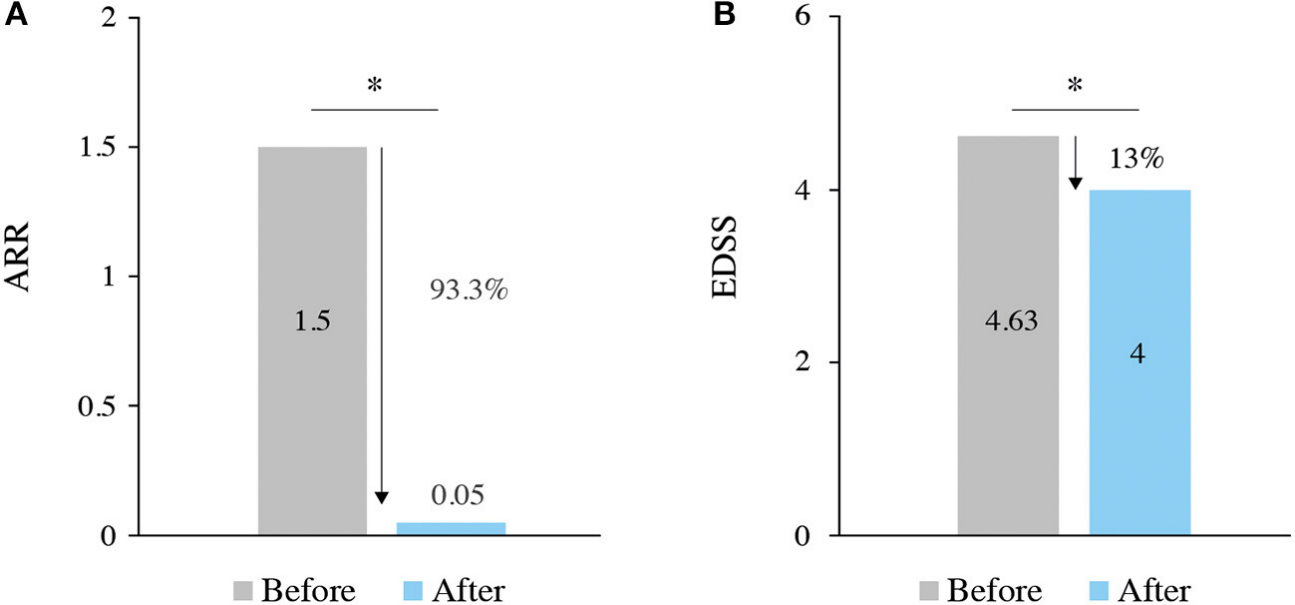
**(A, B)** ARR and EDSS before and after alemtuzumab treatment. ^*^p < 0.001.

Patients were treated with ALZ for a mean of 29.7 (13.8) months. During this period, ALZ significantly reduced the ARR from 1.5 before to 0.05 (95% CI 0.03–0.08) after ALZ (*p* < 0.001) (Figure 1A).

**Figure 2:**
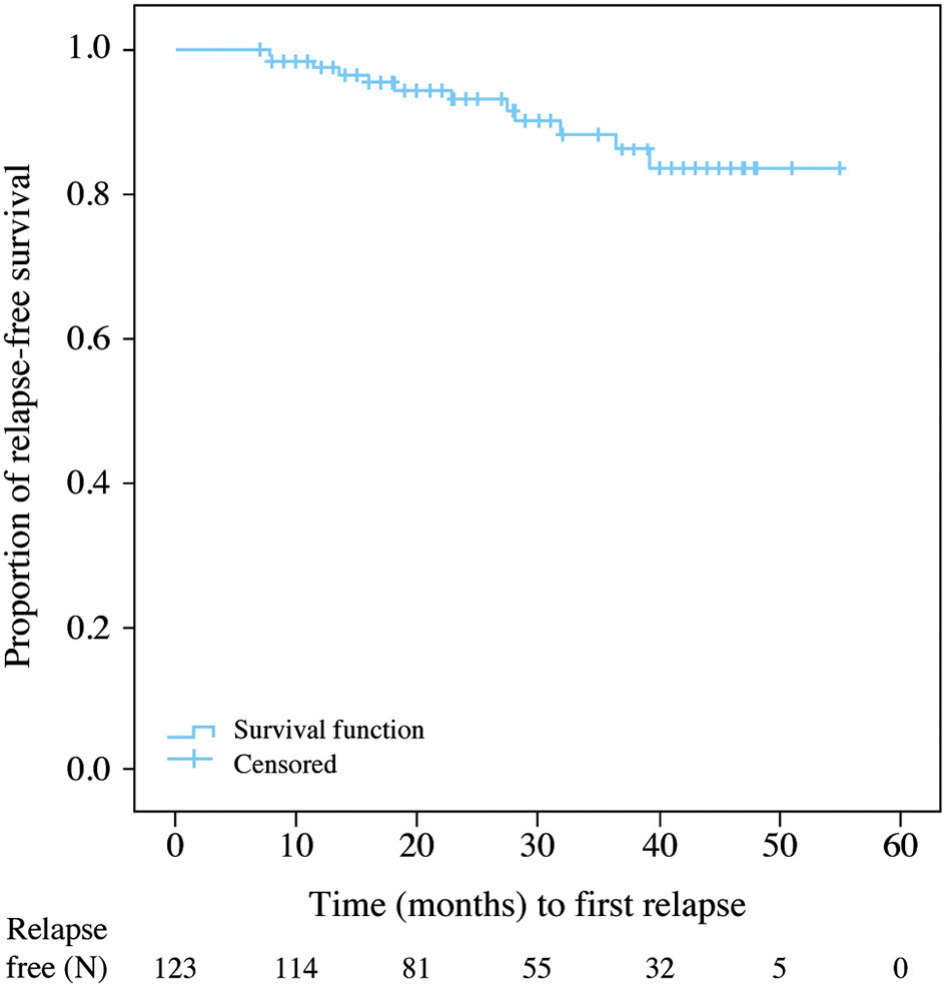
Kaplan–Meier estimates of time to first relapse during alemtuzumab treatment.

A total of 111 (90.2%; 95% CI 83.6–94.9) patients were relapse-free after ALZ and up to the time of data cutoff. Kaplan–Meier estimates showed that the mean time from ALZ initiation to first relapse was 50.1 (CI 47.6–52.7) months (Figure 2).

**Figure 3:**
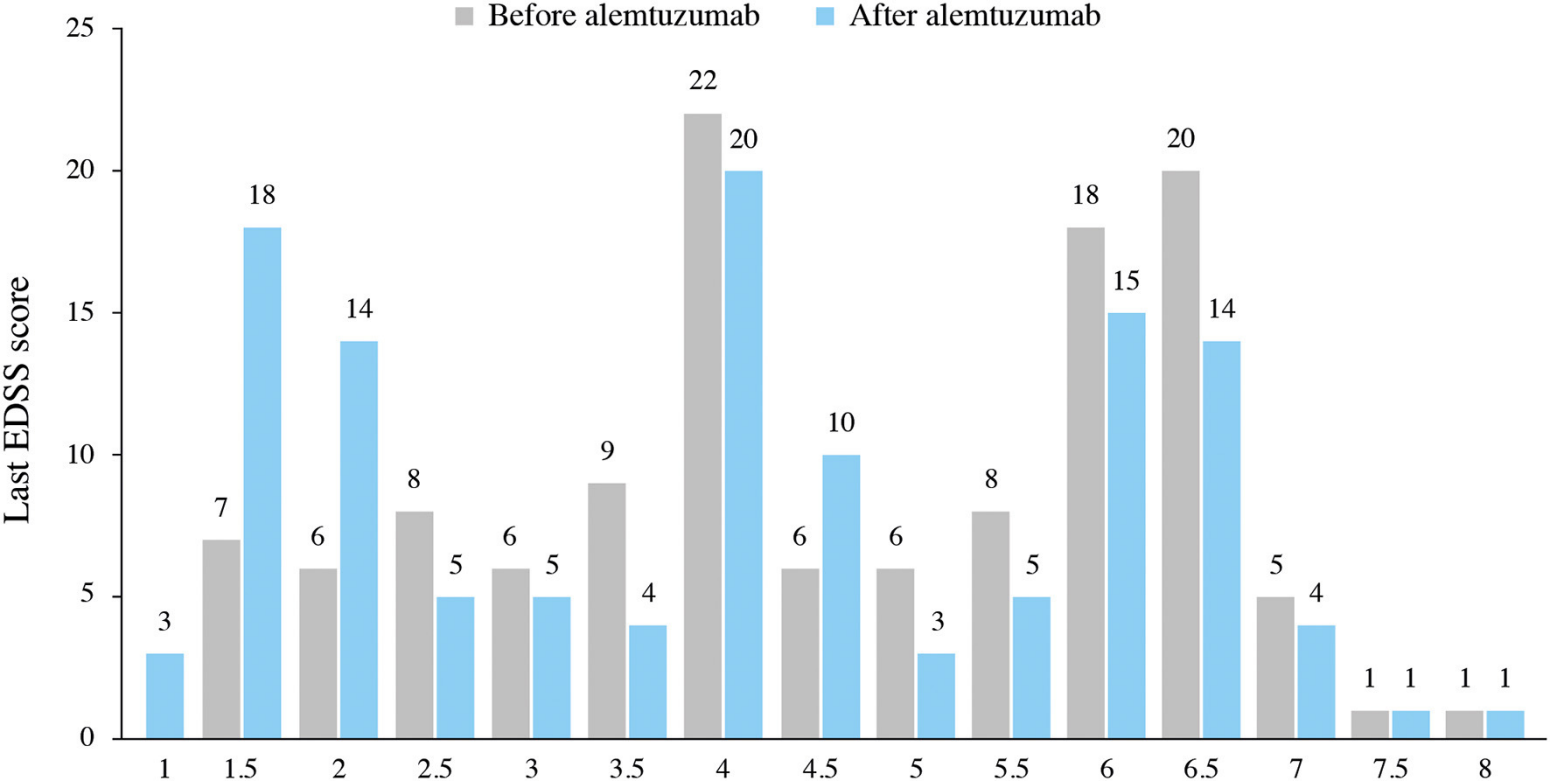
Distribution of EDSS categories.

As displayed in Figure 1B, the median EDSS score significantly improved from the baseline (4.63 before vs. 4.00 after ALZ; *p* < 0.001). The last available EDSS score was ≤ 3 in 21.6% of patients before ALZ, and this percentage of patients increased to 37.4% after ALZ (Figure 3 displays the distribution of EDSS categories before and after treatment). After ALZ initiation, 119 (96.7%) patients were free of 6-month CDW, and 60 (48.8%) patients achieved 6-month CDI.

**Figure 4:**
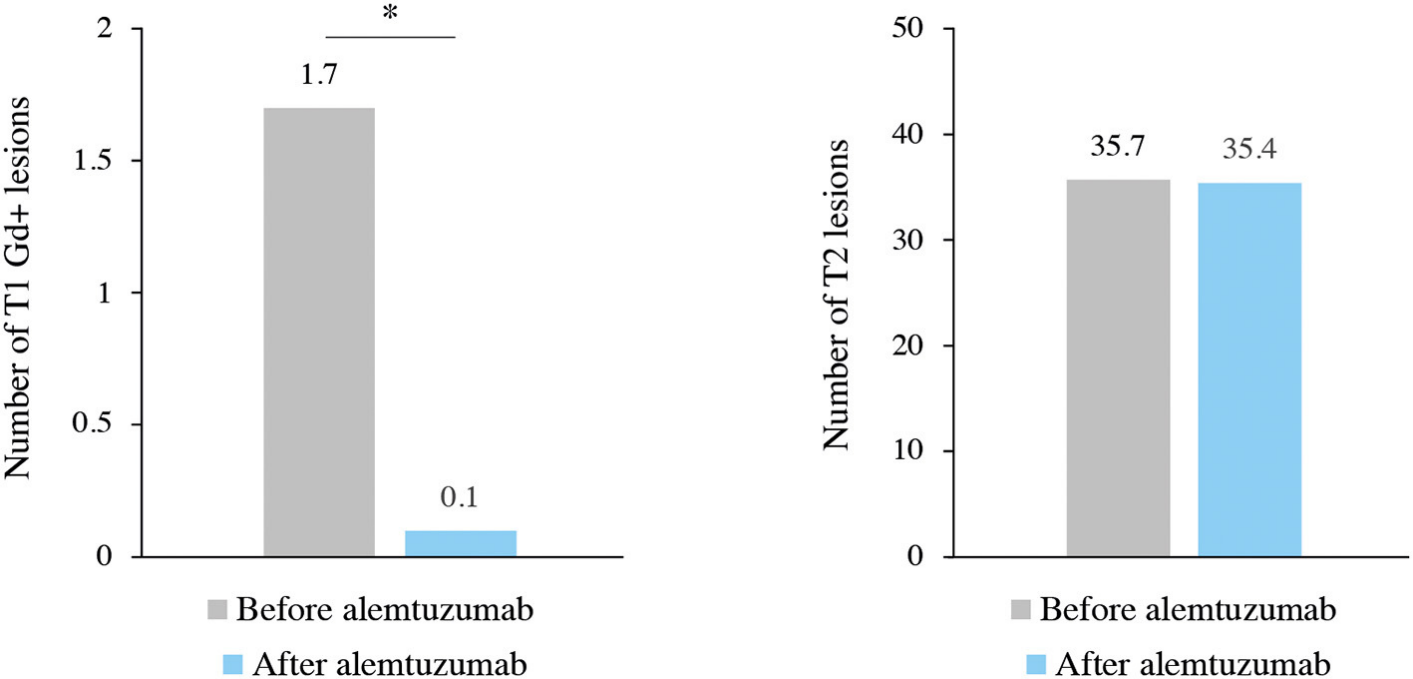
MRI lesion activity before and after alemtuzumab treatment. *p < 0.001.

MRI data of Gd+ T1 lesions and T2 hyperintense lesions were available for 119 and 120 patients, respectively. The mean number of Gd + T1 lesions was significantly reduced (1.7 before vs. 0.1 after ALZ; *p* < 0.001), and the mean number of T2 hyperintense lesions was maintained (35.7 before vs. 35.4 after ALZ; *p* = 0.392) (Figure 4).

### Safety

A total of 97 (78.9%) patients presented IARs after the first ALZ course and decreased with subsequent courses. Two IARs led to the discontinuation of ALZ (alithiasic cholecystitis and abdominal pain with a self-limited renal function alteration that was resolved spontaneously after ALZ discontinuation). The rest of the IARs were graded as mild-moderate, with quick resolutions after either slowing the ALZ infusion rate or supplemental doses of paracetamol, steroid, or antihistamines. Forty-five (36.6%) patients had an infection. The most common infections (in descending order of frequency) were urinary tract infections, nasopharyngitis, gastroenteritis, candidiasis, oral herpes infection, and upper respiratory tract infection. A total of 27 patients (21.9%) reported 29 autoimmune diseases: hyperthyroidism (12), hypothyroidism (11), idiopathic thrombocytopenic purpura (ITP) (3), alopecia areata (1), chronic urticaria (1), and vitiligo (1). A total of 25 patients had one autoimmune disease and two patients had two (one patient had two ITP and one patient had alopecia areata and chronic urticaria). The three episodes of ITP completely resolved in the two patients with oral corticoids. These patients were diagnosed on the basis of routine analytical findings and did not bleed or require blood transfusions or hospitalization.

### Healthcare resource utilization

**Figure 5:**
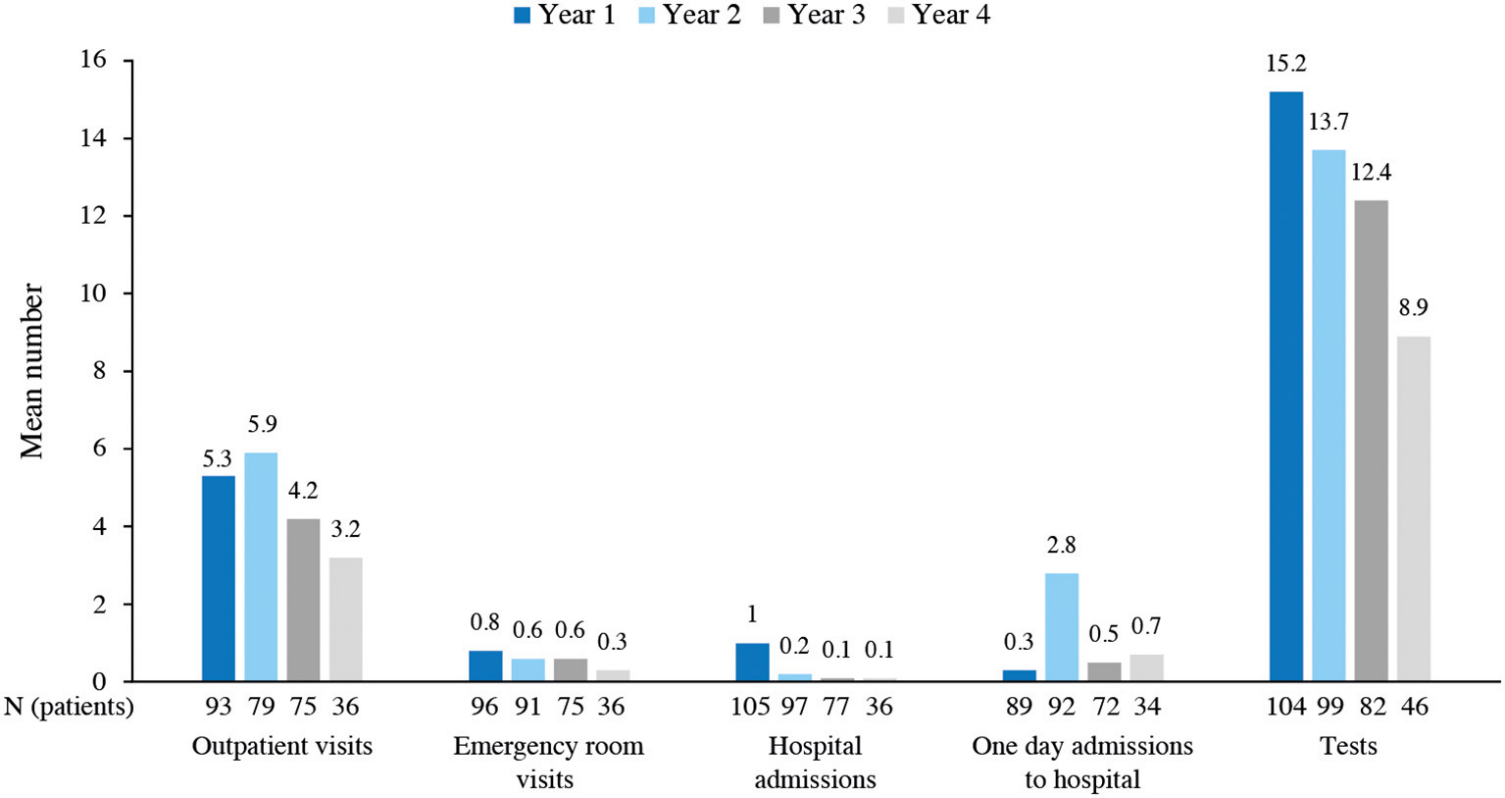
Healthcare resource use during alemtuzumab treatment.

The mean number of outpatient visits, emergency room visits, hospital admissions, and tests performed in the hospital while patients were treated with ALZ progressively decreased from year 1 to year 4 (except for a slight increase in year 2 of outpatient visits). The mean number of one-day admissions to the hospital was overall low but slightly increased from year 1 to year 4, showing a peak at year 2 (Figure 5).

The mean (SD) duration of hospital admissions was reduced by half from year 1 to year 3 (6.4 [2.2] days in year 1, 4.1 [3.0] days in year 2, and 3.1 [1.1] days in year 3) and increased to 4.0 (3.4) days in year 4. As shown in Table 2, most patients received other treatments related to MS (in addition to ALZ) throughout the years.

**Table 2:** Treatments administered more frequently (>10% patients) per year.

	**Year 1**	**Year 2**	**Year 3**	**Year 4**
Patients who received any treatment, *n* (%)	105 (85.4)	100 (81.3)	81 (65.9)	45 (36.6)
Patients who did not receive treatment, *n* (%)	0	1 (0.8)	2 (1.6)	3 (2.4)
Patients with missing data, *n* (%)	18 (14.6)	22 (17.9)	40 (32.5)	70 (56.9)
**Treatments**, ***n*** (%)^*^				
Aciclovir	103 (98.1)	88 (88)	10 (12.3)	8 (18.2)
Dexchlorpheniramine	100 (95.2)	90 (90)	9 (11.1)	6 (13.6)
Paracetamol	94 (89.5)	91 (91)	14 (17.3)	7 (15.9)
Baclofen	29 (27.6)	27 (27)	19 (23.5)	10 (22.7)
Fampridine	22 (21.0)	21 (21)	21 (25.9)	9 (20.5)
Gabapentin	20 (19.0)	13 (13)	15 (18.5)	7 (15.9)
Amitriptyline	19 (18.1)	17 (17)	15 (18.5)	7 (15.9)
Omeprazole	18 (17.1)	13 (13)	10 (12.3)	3 (6.8)
Methylprednisolone	15 (14.3)	17 (17)	7 (8.6)	3 (6.8)
Citalopram	13 (12.4)	10 (10)	8 (9.9)	6 (13.6)
Tamsulosin	12 (12.4)	14 (14)	6 (7.4)	3 (6.8)
Pantoprazole	12 (12.4)	12 (12)	1 (1.2)	1 (2.3)
Lorazepam	11 (10.5)	12 (12)	7 (8.6)	3 (6.8)
Diazepam	11 (10.5)	6 (6)	7 (8.6)	4 (9.1)
Levothyroxine sodium	4 (3.8)	13 (13)	18 (22.2)	14 (31.8)
Duloxetine	10 (9.5)	11 (11)	6 (7.4)	2 (4.5)
Mirabegron	7 (6.7)	11 (11)	8 (9.9)	3 (6.8)

* Valid percentages are shown here (i.e., percentages calculated considering the total number of patients receiving other treatments).

## Discussion

The ReaLMS was a retrospective study that confirmed the effectiveness and safety of ALZ for MS treatment in a sample of 123 patients and provided information regarding the healthcare resource use by these individuals in one Spanish center. Compared with the baseline characteristics from the pivotal trials (5, patients in this study were older (ReaLMS, 40.3 years; CARE-MS I, 33 years; CARE-MS II, 34.8 years), had a longer disease duration (ReaLMS, 13.8 years; CARE-MS I, 2.1 years; CARE-MS II, 4.5 years), a higher EDSS score (ReaLMS, 4.5; CARE-MS I, 2.0; CARE-MS II, 2.7), and were treated with a higher number of previous DMTs (ReaLMS, 78.8% received ≥2 previous DMTs; CARE-MS I, were treatment naive; CARE-MS II, 18% received ≥2 previous DMTs). Overall, our study showed that patients with MS treated with ALZ under clinical practice conditions in Spain had more advanced diseases than patients in the pivotal trials. This difference in the patient profile between clinical trials and observational studies has been recently documented. A study that included 3,577 patients with MS showed that only one-fifth of patients treated with ALZ in routine clinical practice would have met the selection criteria for the phase III clinical trials (24). The interim analysis of the LEMVIDA study in Spain also showed that patients were at a later disease stage, had a greater disability, and had received strong immunotherapies compared to patients in the pivotal trials (12). The above-mentioned studies along with our findings reinforce the importance of conducting studies in patients treated with ALZ under real-world conditions to complement the body of evidence from clinical trials.

Alemtuzumab-reduced clinical and radiological disease activity is in line with previous results from clinical trials (5, 6, 25) and from observational studies (15, 18, 26–28). The percentage of relapse-free patients after ALZ treatment in the ReaLMS (90.2%) was higher than in previous studies (75–84.8%) (18, 26). We also observed that ALZ decreased EDSS scores, as previously reported (15, 18, 27, 29). Almost all our patients (96.7%) were free of 6-month CDW when the study data were cut off. Previous studies observed that this percentage remained considerably high through 36 months (82%) (18) and up to 7 years (59.8%) (20) and 9 years (62%) (30), emphasizing the positive effect of ALZ on long-term disability. However, as shown by a recent study, receiving ALZ as a third-line treatment increases the probability of relapses and disability worsening compared with receiving it as the first or second-line therapy, a phenomenon that is more pronounced in patients previously treated with fingolimod (29). Considering that most patients in our study received ALZ as a third-line DMT or after fingolimod, the reduction of clinical and radiological activity and the disability improvements and stabilization observed after ALZ treatment in our study is, indeed, remarkable.

Importantly, all patients in the study were treated before the ALZ label was updated. Although clinicians had more flexibility to start ALZ treatment before the label changed (11), the present study shows that in our hospital, most of the patients initiated ALZ after the failure of the previous DMT (ALZ was chosen as a first DMT only in 9% of the patients). This therapeutic approach is in line with the current ALZ label and with the recommendations provided by the Spanish expert consensus (31). However, the debate on whether to select for the first treatment after diagnosis a lower-efficacy but relatively safe DMT (escalation approach) or a high-efficacy DMT is still ongoing. Findings from retrospective studies have already suggested that in patients with disease activity, the early use of high-efficacy DMT may delay disability progression (32, 33) and increase the likelihood of achieving NEDA (34) to a higher extent than moderate efficacy DMT. The European (ECTRIMS/EAN) guidelines do not advocate for any of the two possible treatment approaches but state that the DMT choice should be based on the characteristics of the patient, the disease activity, and the safety profile and accessibility of the DMT (35). Ongoing randomized clinical trials evaluating these treatment approaches (TREAT-MS, NCT035300328; DELIVER-MS, NCT03535298) together with the collection of individual clinical data and computational advances will allow gathering further evidence to improve the individualized DMT choice (36, 37).

The safety outcomes observed in our study are similar to those reported in the pivotal trials (5, 6, 9) and other real-world evidence studies (19, 38). Approximately three-quarters of patients had IARs after the first cycle, but the percentage of IARs decreased with successive ALZ courses. A total of two out of 123 treated patients had IARs that led to the discontinuation of ALZ, which was similar to the 1% discontinuation rate due to AEs from the CARE-MS I (5) and lowering to the 3% discontinuation rate from the CARE-MS II (6). Although 11.6% of patients had been previously treated with natalizumab, no case of PML was detected. No severe herpes viral infections, nocardiosis, or listeriosis, previously reported as rare infections (37) occurred until the study data were cut off. Two patients developed ITP, an AE that was previously observed during the pivotal trials (5, 6). The presence of autoimmune disorders suggests that there is an immune dysregulation probably caused by a differential lymphocyte repopulation following ALZ. In line with the presence of AEs and IARs after ALZ, close monitoring is highly recommended.

Most of our patients received a total of two ALZ courses. The percentage of patients who needed more than two courses until study data were collected (6.5%) was lower than in the pivotal trials (36%) (7) and other observational studies (40%) (28). A study conducted in the Netherlands showed that the decreasing number of patients requiring further ALZ treatment resulted in cost savings compared to fingolimod and natalizumab for approximately 3 years after treatment initiation (39). The key driver of these cost savings was the cumulative difference in treatment costs due to the low number of ALZ courses that patients require within 5 years (39). Similar conclusions were drawn from a study conducted in Spain that also considered a time horizon of 5 years, showing that ALZ, compared with fingolimod and natalizumab, resulted in an estimated savings of € 3.7 million for treating 100 patients in the Spanish National Health System (40). The increase in the number of 1-day admissions to the hospital showed a peak in year 2 and was probably due to the administration of ALZ at the day hospital in year 2. Similarly, the duration of hospital admissions in year 1 was the highest likely due to the administration of treatment. Nevertheless, hospital admissions remained low throughout the observation period, which is a favorable result.

We are aware that the ReaLMS suffers from several limitations, intrinsic to retrospective studies, and the interpretation of our findings should be taken cautiously. First, information was obtained from the medical record, which was recorded for clinical purposes and not for research purposes; evaluations were not standardized, likely resulting in underreported outcomes and missing data. Not all adverse events were recorded using the same terminology or classification system per clinical practice, which might have altered the accuracy of these data. Although this can be considered a limitation of the study, it should be noted that the description of AEs due to ALZ was a secondary objective of the present study. Second, data were obtained from a tertiary center, and the findings cannot be generalized to the common clinical practice in Spain. Third, although patients with comorbidities were not excluded, the analysis of the effectiveness and safety in patients with a genetic predisposition to specific complications such as susceptibility to vascular events or autoimmune disorders were not conducted, and therefore, the generalization of our findings to these populations might be also limited. Another limitation of the study was the lack of a comparison group, including patients with MS receiving a different DMT at the study center, which did not allow us to compare treatment outcomes and healthcare resources used throughout the years with another group. Moreover, costs associated with healthcare resource use were not collected here, and therefore, we could not provide economic data, which would have been of interest. Finally, we did not analyze ALZ's effectiveness and safety and the use of the healthcare resource according to previous treatment. This and other baseline characteristics should be taken into consideration in future studies as they provide useful information to support treatment decisions in patients with MS.

## Conclusion

The ReaLMS study provides real-world evidence that ALZ can promote clinical and MRI disease remission (Gd + lesions) as well as disability improvement (EDSS) in patients with MS despite failure with multiple previous DMT. The ALZ safety profile was consistent with data available from clinical trials and other real-world studies, that did not raise new safety concerns and had a more favorable safety profile than in some prior studies. Healthcare resource use decreased from 1 to 4 years. Future studies evaluating the budget impact of ALZ for treating patients with MS in Spain are warranted.

## Data availability statement

The raw data supporting the conclusions of this article will be made available by the authors on request to the corresponding author.

## Ethics statement

The studies involving human participants were reviewed and approved by the Ethics Committee of the Hospital Universitario Virgen Macarena. The patients/participants provided their written informed consent to participate in this study.

## Author contributions

SE: conceptualization, data curation, formal analysis, funding acquisition, investigation, methodology, project administration, resources, software, supervision, validation, visualization, writing—original draft, and writing—review and editing. RLR: conceptualization, data curation, formal analysis, investigation, methodology, project administration, supervision, validation, visualization, and writing—review and editing. MÁC and GN: supervision, validation, and writing—review and editing. MRA: conceptualization, data curation, supervision, and writing—review and editing. JR-P: investigation, supervision, validation, and writing—review and editing. JLMA: methodology and writing—review and editing. JD: data curation, investigation, methodology, supervision, validation, visualization, and writing—review and editing.
